# Exploring the Effectiveness of an Integrated Physical Activity and Psychosocial Program Targeting At-Risk Adolescent Girls: Protocol for the Girls United and on the Move (GUM) Intervention Study

**DOI:** 10.2196/15302

**Published:** 2020-06-09

**Authors:** Cristina M Caperchione, Nicole Hargreaves, Catherine M Sabiston, Stephen Berg, Kent C Kowalski, Leah J Ferguson

**Affiliations:** 1 School of Sport, Exercise and Rehabilitation University of Technology Sydney Sydney Australia; 2 School of Health and Exercise Science University of British Columbia Okanagan Kelowna, BC Canada; 3 Faculty of Kinesiology & Physical Education University of Toronto Toronto, ON Canada; 4 Okanagan School of Education University of British Columbia Okanagan Kelowna, BC Canada; 5 College of Kinesiology University of Saskatchewan Saskatoon, SK Canada

**Keywords:** adolescence, girls, at-risk, self-compassion, sport enjoyment, physical activity, community-based intervention

## Abstract

**Background:**

Adolescents are highly susceptible to negative self-perceptions, likely due to their social cues and environment. The presence of these negative self-perceptions has been shown to adversely impact levels of physical activity (PA). Although PA has the ability to foster improved self-perceptions, the rates of PA among adolescents continue to descend, with girls appearing to be most susceptible to these declines. At-risk adolescent girls, who may experience a number of negative preceding lifestyle conditions, may be exceptionally vulnerable to declines in PA. There are a high number of adolescent girls from low-income and abusive households in British Columbia, Canada, thus indicating a need for a program to relay the importance of PA and healthy lifestyle behaviors.

**Objective:**

This paper aims to describe the protocol of the Girls United and on the Move (GUM) pragmatic intervention, an integrated PA and psychosocial program aimed at improving self-compassion, social connectedness, and overall self-perceptions among at-risk adolescent girls.

**Methods:**

Using a quasi-experimental mixed methods approach, the GUM intervention was conducted in 5 schools in British Columbia, Canada. Adolescent girls aged 11 to 15 years who were identified as at risk were included in the study. The 9-week intervention, co-delivered by a PA/health promotion–trained researcher and a registered social worker, involved a PA component and a psychosocial component with evidence-based topics addressing the concerns of the adolescent girls. The following outcomes were evaluated: PA, self-compassion, social support, leader supportiveness, and sport enjoyment and commitment. Program acceptability and satisfaction was also examined. Outcome measures were assessed at baseline (week 1), week 6, and postintervention (week 9), and interview data concerning program acceptability and satisfaction were collected at postintervention from a subsample of participants.

**Results:**

A total of 101 participants were invited to participate in the GUM intervention. Reporting of the results is projected for the fall of 2020.

**Conclusions:**

It is anticipated that the GUM intervention will enhance PA while also improving self-compassion, social connectedness, and overall self-perceptions among at-risk adolescent girls. The findings of this research will contribute to the literature concerning PA and various psychosocial factors that impact the physical and mental health of at-risk adolescent girls.

**Trial Registration:**

Clinicaltrials.gov NCT03567200; https://clinicaltrials.gov/ct2/show/NCT03567200.

**International Registered Report Identifier (IRRID):**

DERR1-10.2196/15302

## Introduction

Worldwide levels of physical activity (PA) among adolescent girls have seen a substantial decline in past decades [1,2], with this decline being related to a number of physical and mental health issues (eg, increased risk of cardiovascular diseases, overweight and obesity, type 2 diabetes, depression, anxiety, poor emotional health, and overall mortality rates) [3-5]. Despite enhanced efforts to increase levels of PA among adolescent girls, only 33% of adolescent girls currently report being physically active, with only 16% meeting the PA recommendations put forth by the World Health Organization [6] (ie, a minimum of 60 minutes of moderate-vigorous intensity PA daily). The lack of participation in PA and sports among adolescent girls is thought to be influenced by a number of negative self-perceptions, including low levels of self-compassion, poor social support, and social isolation [7-10].

At-risk adolescent girls are especially vulnerable to these negative self-perceptions, as they may be faced with additional hardships, including low socioeconomic housing, exposure to substance abuse, sexual exploitation, etc [11]. At-risk adolescents have been known to have a higher prevalence of depression and low self-esteem compared with the general population of adolescents [9], possibly leading to a higher likelihood of adopting future problem behaviors, which consequently have more serious long-term consequences [12,13]. In fact, conditions such as low income, insufficient caregiving, and family breakup have all been identified as conditions predictive of youth delinquency [14,15]. Adolescents who are categorized as at risk in this regard are also at higher risk of compromised health outcomes, such as obesity, cardiovascular diseases, type 2 diabetes, and numerous other chronic diseases [2]. For the purposes of this study, at risk was operationally defined as one with a dysfunctional family life, socioeconomic instabilities, various forms of abuse, or mental health issues such as anxiety or depression [16,17].

By providing an emotionally supportive environment for this population, it is likely that enhanced physical and mental health outcomes will be exhibited, as a strong support system is able to establish a sense of belonging and aid in case of problems [18]. In particular, engaging in regular PA during adolescence has been directly linked to reduced rates of anxiety, depression, emotional disturbances, and psychological distress [19,20] and improved self-perception facets, such as self-esteem, self-confidence, self-compassion, and perceived social support [20-22]. For instance, Raudsepp and Vink [23] examined the longitudinal associations between girls’ sedentary behavior and depressive symptoms and found that higher levels of depressive symptoms were predictive of greater sedentary behavior. Additionally, Kliziene and colleagues [24] investigated the influence of a 7-month exercise intervention on levels of anxiety among adolescents aged 14 to 15 years and revealed that the exercise intervention was related to decreased anxiety scores. Further, self-compassion, or the way in which an individual offers kindness and support to oneself [25], has been shown to increase in individuals who are physically active [26], and by increasing self-compassion, one is more apt to engage in other healthful behaviors (eg, healthy diet, stress-reducing practices) [27]. Social support provided from peers and significant adult figures has been shown to play a large role in the adoption of various health behaviors, and the critical role that program leaders play as personal role models has been vastly highlighted throughout the literature [28,29]. Positive self-perceptions and PA and sport enjoyment are also key emotional and motivational processes that have been linked to continued participation in PA [30].

There have been various interventions to date with the goal of improving PA behavior among adolescent girls; however, few have focused on at-risk girls. There is a need for tailored and targeted evidence-based programs focused on addressing specific physical, mental, and psychosocial health issues that at-risk adolescent girls may currently face in everyday life. To address these issues, Girls United and on the Move (GUM), an integrated PA and psychosocial program, was developed. The overarching purpose of the GUM study was to explore the effectiveness of an integrated PA and psychosocial program on PA behaviors and various identified psychosocial factors among at-risk adolescent girls. The purpose of this paper is to describe the intervention design and methodological protocol of the GUM program.

## Methods

### Study Design

**Figure 1:**
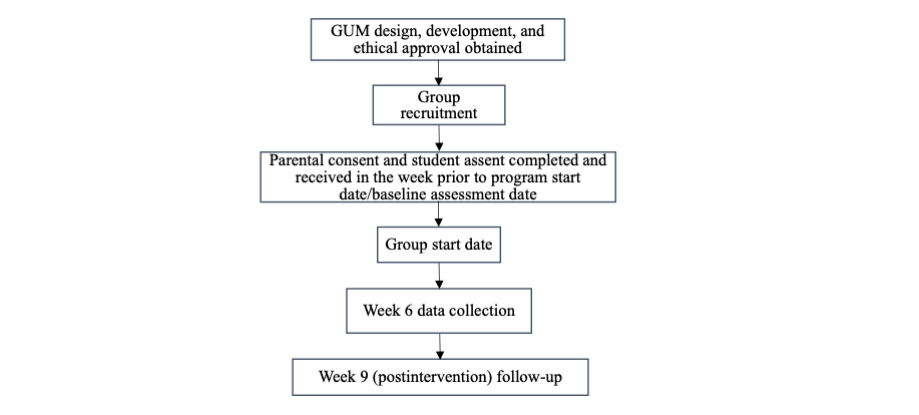
Flow diagram of the GUM protocol. GUM: Girls United and on the Move.

The GUM study protocol was prepared according to the Standard Protocol Items: Recommendations for Interventional Trials (SPIRIT) guidelines [31] and used an exploratory quasi-experimental pretest-posttest design. Data collection occurred at baseline (T1), week 6 (T2), and postintervention (T3) and included self-report questionnaires and semistructured interviews. Recruitment for the GUM program began in December 2017 and was completed in May 2019. Figure 1 provides a flow diagram of the GUM protocol, and Table 1 provides a detailed description of relative program recruitment dates.

**Table 1:** Detailed description of relative program start dates^a^.

Group No.	Number of participants	Program recruitment
1	13	January 2018
2	11	January 2018
3	13	April 2018
4	9	April 2018
5	7	July 2018
6	14	September 2018
7	10	September 2018
8	11	October 2018
9	13	May 2019

^a^Program commencement dates were consistent with program recruitment dates. Each program was 9 weeks in duration.

### Ethical Approval

Ethical approval was obtained from the Behavioural Human Research Ethics Board at the University of British Columbia (H17-01540) and from School District 23. In all cases, both parental consent and student assent were obtained prior to data collection.

### Objectives

The specific objectives of the GUM study were (1) deliver and explore the effectiveness of the 9-week GUM intervention program on the primary outcomes of PA, self-compassion, social support, and leader behavior; (2) explore the effectiveness of the 9-week GUM intervention program on the secondary outcomes of sport enjoyment and commitment; and (3) examine participant perceptions of the GUM program in terms of acceptability and satisfaction.

### Participants, Setting, and Recruitment

Participants of the GUM study included adolescent girls from 5 middle schools in British Columbia, Canada, who were aged 11 to 15 years and identified as at risk. Guidance counselors from each middle school used these criteria to identify girls and invite them to participate, encouraging those whom they felt would benefit from participating in the program. The guidance counselors were exclusively responsible for recruitment because they had previous history working with the girls on a regular one-to-one basis due to the girls’ behavioral inadequacies, issues with home life, or mental health–related concerns such as anxiety or depression. Counselors then provided interested individuals with further detailed information about the program (including parental consent and student assent forms) and prompted the students to discuss the program with their parents or guardians. Participants were asked to return the signed consent and assent forms to the guidance counselor or the research team prior to the start of the program or at T1. There were no specific exclusion criteria except that participants could not have been a participant of the previous pilot study, Girls on the Move (GoM) which took place from 2015 to 2016 [32]. A total of 101 participants forming 9 groups were recruited from 5 schools throughout the region, with group sizes ranging from 7 to 14 participants per school. Each group had a different start date, with some groups in different schools running simultaneously. However, no two groups from the same school received the intervention at the same time. Recruitment was completed in May 2019.

Each GUM intervention session occurred at the participating school (eg, a classroom, gymnasium, or outside on school grounds) or off campus within the community, depending on the scheduled activity for the given day (eg, kickboxing occurred at a local community kickboxing and martial arts studio). The participants were provided with transportation to all off-campus activities by the trained researcher and social worker (ie, program facilitators). Both facilitators were authorized and insured to provide transportation for program participants. Participation was completely voluntary and all participants were informed that they did not need to participate in the research component to participate in the GUM program. Those who chose to participate in the research component were informed that they could withdraw from participation in the study at any time and without consequence.

### Description of the GUM Intervention

#### Pilot Study

The GUM intervention was developed and refined based on the GoM pilot study. GoM was conducted from 2015 to 2016 and included 2 middle schools, with a total of 24 participants aged 11 to 17 years. The purpose of the GoM intervention was to examine program and methodological feasibility and gain further insight concerning PA experiences, preferences, and self-concept among at-risk adolescent girls [32]. Findings from the GoM program indicated that the assessments used were appropriate, program delivery (ie, duration, geographic setting, and facilitation) was acceptable, and participants were satisfied with the activities and content. The findings also suggested that adolescent girls are aware of the importance of proper PA and nutrition in relation to health outcomes, believe that peer social support is important for PA engagement, and feel that enjoyment is crucial when dictating PA participation. Based on these outcomes, minor refinements were made to the GUM intervention protocol and research methodology, including the addition of alternative physical activities on and off school grounds, the use of personalized journals for participants to write in, the addition of measures to assess different contexts of social support and the impact of the leaders and facilitators, and the name change from Girls on the Move to Girls United on the Move.

#### Program Components

The GUM intervention was a pragmatic PA and psychosocial program designed specifically for at-risk adolescent girls. Components of the self-determination theory, particularly autonomy, competence, and relatedness, were integrated throughout the intervention because these are known to affect intrinsic motivation and engagement in PA, especially among underserved youth [11,33]. The GUM intervention consisted of 9 weekly, group-based, 90-minute face-to-face sessions that were cofacilitated by a trained researcher and a registered social worker from a partnering community organization or key stakeholder. The partnering stakeholder was a nonprofit organization with the goal of bringing an end to violence and poverty and seeking justice for children and women. This nonprofit organization works extensively with adolescent populations, particularly those categorized as at risk. Each GUM group consisted of 7 to 14 at-risk adolescent girls from participating schools. Smaller groups were desired in order to create an intimate and supportive environment and provide a greater opportunity for participants to get acquainted with each other and the program facilitators.

Within each of the weekly 90-minute program sessions, 45 minutes of PA were delivered by a trained researcher and 45 minutes were allocated to social worker–led discussions on various psychosocial topics. Examples of physical/sport activities included on-campus activities such as yoga, team obstacle courses, and soccer, or off-campus activities such as rock climbing, self-defense classes, and fitness classes. Examples of psychosocial topics that were discussed within each group included healthy relationships, conflict resolution strategies, substance abuse issues, sexuality, and gender issues. Table 2 outlines the components of the GUM intervention.

In addition to the group discussions facilitated by the social worker, personal journals were distributed to each participant during week 1. These journals provided participants with an opportunity to disclose their thoughts and feelings about their current state, any personal struggles they may be undergoing, or anything to do with the GUM program itself. Participants returned the journals to the registered social worker at the end of each of the weekly GUM sessions, indicating if they wanted the social worker to read the entry and give a written response. This allowed participants to reach out and seek advice if they needed it, without requiring the participants to talk in person about their issues to either one of the program facilitators. Although not previously used in the GoM pilot study, disclosure journals have been used previously by the social worker and have been reported to be an important component of previous programs delivered to this population. The disclosure journals were used as a component of the intervention, but no data were extracted or evaluated from the journals. After program completion (ie, after week 9), a subsample of participants (30/101, 29.7%) were invited to participate in an individual semistructured interview so that the program facilitators could gain further information concerning program acceptability and satisfaction. Those who attended a minimum of 80% (7/9) of the program sessions were invited to participate in the voluntary semistructured interviews. Participants were informed that they were able to withdraw from the study at any time, for any reason, without consequence. All personal data were coded and handled with confidentiality.

**Table 2:** Components of the Girls United and on the Move (GUM) program.

Week	Physical activity component	Psychosocial component
Week 1 (T1)^a^	N/A^b^	N/A
Week 2	Yoga	Emotional wellness
Week 3	Dance	Self-awareness, self-esteem, and body image
Week 4	Self-defense	Healthy relationships
Week 5	Hike/walk outdoors	Healthy sexuality
Week 6 (T2)^c^	Rock climbing	Communication skills, conflict resolution, and boundaries
Week 7	Kickboxing	Sexual exploitation and abuse
Week 8	Free play and outdoor games (eg, capture the flag, scavenger hunt)	Media and gender issues
Week 9 (T3)^d^	N/A	N/A

^a^Introductions and questionnaires were completed. Baseline data were collected at T1.

^b^N/A: not applicable.

^c^The Learning Climate Questionnaire was the only self-report questionnaire administered at T2. See Outcome Measures for further information.

^d^Questionnaires and a wrap-up were completed. Follow-up data were collected at T3.

### Outcome Measures

**Figure 2:**
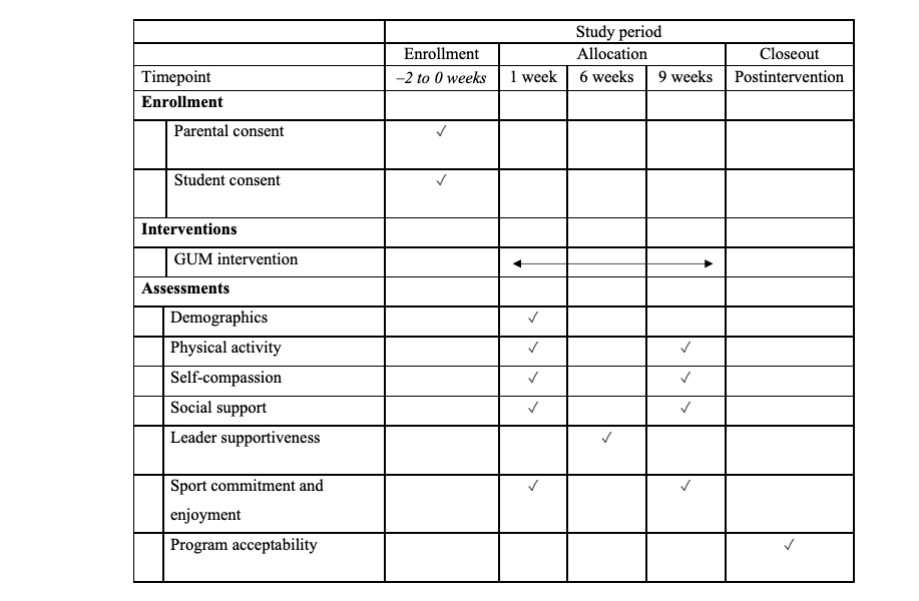
Standard Protocol Items: Recommendations for Interventional Trials figure for the GUM study. GUM: Girls United and on the Move.

Mixed methods were used to collect and analyze data. Quantitative data measures consisted of self-report questionnaires to assess self-compassion, social support, leader supportiveness, and sport commitment and enjoyment. Qualitative data consisted of semistructured interviews with a subsample of program participants, which provided further insight concerning the acceptability and satisfaction of the program components, physical and psychosocial activities, resources, and overall delivery (ie, environment/facilities, facilitators, etc). Self-report measures were used because they are easy to administer, relatively inexpensive, and recognized as an acceptable means of recording participant responses and psychological constructs in youth populations [34]. Further, the guidance counselors advised us that handing out wearable measurement devices, such as accelerometers and pedometers, and having participants return them after the program could be very difficult, with a high likelihood that the devices would be lost, damaged, or stolen, which the counselors identified as an unfortunate reality within an at-risk population. The majority of the assessments occurred at T1 and at T3 at each participating school. Demographic information was collected during T1 and the Learning Climate Questionnaire (LCQ) [35] was completed at T2. Figure 2 provides the SPIRIT figure for the GUM trial.

#### Demographics

Demographic information consisted of age, grade, number of siblings, housing location, mode of transportation to school, and whether the participant took part in any PA inside or outside of school.

#### Physical Activity

Level of PA was assessed using the Physical Activity Questionnaire for Children, a 10-item questionnaire that assesses the frequency of PA participation over a 7-day recall period [36]. This assessment has been reported to be a valid and reliable measure of PA among children and adolescents as it is able to accurately measure general PA patterns regarding intensity, frequency, and duration [36].

#### Self-compassion

The Self-Compassion Scale (SCS) was used to assess self-compassion through 6 subscales that include contrasting components: self-kindness vs self-judgement, mindfulness vs overidentification, and common humanity vs isolation [37,38]. The SCS is a 26-item scale that measures each of these components using a 5-point Likert scale (1=almost never, 5=almost always). The mean scores of each of the subscales can then be combined to yield a total score, which reflects a global self-compassion score [38].

#### Social Support

Perceived social support was assessed using the Child and Adolescent Social Support Scale (CASSS) [39]. The CASSS consists of five 12-item subscales that measure social support received from a variety of sources, including parents, teachers, classmates, close friends, and the school environment [39]. However, a condensed version without the school environment subscale was used, as the actual sources of social support received in the school environment were not well defined and were deemed unnecessary for the purposes of the GUM study [40]. The CASSS measures each of the items on a 6-point Likert scale, ranking each item on how often the participant feels they receive social support from the indicated source, from never (1) to always (6). Additionally, each item has a perceived importance scale, which is measured using a 3-point Likert scale ranging from not important (1) to very important (3). The CASSS has been represented as a valid and reliable measure for its intended purposes [40].

#### Perceived Leader Supportiveness

The LCQ [35] was used to assess participants’ perceptions of support provided by the program facilitators. The LCQ is a 15-item measure with values ranging from strongly disagree (1) to strongly agree (7). It provides a good indication of the support that leaders provided for the 3 psychological needs (ie, autonomy, competence, and relatedness) [35]. Research shows that when these needs are supported, higher levels of psychological well-being are evident [35]. The LCQ has been shown to have extensive internal consistency and reliability, and it provides a valid indication of perceived supportiveness among youth outside of sporting contexts [41]. The LCQ was the only self-report questionnaire that was not administered at both T1 and T3; instead, it was distributed at T2.

#### Sport Commitment and Enjoyment

Sport commitment and enjoyment was assessed using the Sport Commitment Model [42], a questionnaire that has been shown to be a valid and reliable measure of sport commitment and enjoyment among youth [42]. The model addresses the 5 components related to sport commitment, namely sport enjoyment, personal investments in the self-indicated activity, involvement opportunities, social constraints, and involvement alternatives. Each of the 58 items is assessed using a 5-point Likert scale, with scores ranging from strongly disagree (1) to strongly agree (5).

#### Program Acceptability and Satisfaction

Face-to-face, individual, semistructured interviews were conducted with a subsample of participants (30/101, 29.7%) to gain greater knowledge concerning acceptability of and satisfaction with the GUM program. Participants were asked to share their thoughts, opinions, experiences, and recommendations concerning activities, education, and resources of the program. These interviews were conducted at postintervention by a trained researcher at a time that was convenient to the participants. Interview questions were informed by the study objectives and the research literature specific to this topic area and study population. Data from the interviews were audio recorded and transcribed verbatim with all possible identifiable information removed.

To enhance rigor, various verification strategies were used, such as having the data systematically checked by other trained researchers and closely monitoring gradually learned information as the research project progressed. Table 3 provides a summary of the measures used at the various data collection time points.

**Table 3:** Summary of measures and data collection time points.

Measure	Methods for data collection	Data collection time points
Demographics	Age, grade, number of siblings, housing location, main mode of transportation to school, participation in organized PA or sport inside or outside of school	Week 1 (baseline only)
Physical activity	Physical Activity Questionnaire for Children	Weeks 1 and 9
Self-compassion	Self-Compassion Scale	Weeks 1 and 9
Social support	Child and Adolescent Social Support Scale	Weeks 1 and 9
Perceived leader supportiveness	Learning Climate Questionnaire	Week 6 only
Sport commitment and enjoyment	Sport Commitment Model	Weeks 1 and 9
Program acceptability	In-person semistructured interviews	After week 9 (postintervention only)

### Data Analysis

All data collected for descriptive purposes will be presented as means and standard deviations for all sample characteristics. All outcome variables, including changes in PA behavior, self-compassion, social support, perceived leader supportiveness, and sport commitment and enjoyment, will be analyzed using an analysis of covariance statistical design. The level of significance will be set at α=.05. Statistical analyses will be conducted using SPSS software (version 21.0; IBM Corp).

Inductive thematic analysis, as outlined by Braun and Clarke [43], will be used to analyze the interview data. Data analyses will be conducted by 2 trained researchers who will independently read the transcripts numerous times to become familiar with the data and generate initial codes by identifying important features of the data that are relevant to the study objective. Coded data will then be examined for similarities, grouped, and refined to identify potential themes [43]. Representative quotes will be used to provide evidence of the themes within the data.

### Data Management

Data collection, as well as the handling and storage of data, will be coordinated within the Physical Health and Activity Behaviour (PHAB) Lab at the University of British Columbia. Demographic and self-report questionnaire data will be entered electronically by a trained researcher on the project. All paper-based data will be stored in a secure and locked filing cabinet located in the PHAB Lab. All electronic data will be stored on a password-protected computer also located in the PHAB Lab.

## Results

The final recruitment phase for the GUM study was completed in May 2019, with the final study results projected to be published in the fall of 2020. The results of the GUM study will be disseminated through traditional avenues such as presentations at national and international academic meetings and publication in open-access, peer-reviewed journals. In addition, these results will be disseminated through plain language summaries to participants and through summary briefings to local stakeholders and government agencies.

## Discussion

This protocol paper describes the methodology processes of a unique intervention encompassing both PA and psychosocial components, designed to help address the specific issues and barriers faced by an underserved subpopulation of adolescent girls. Through evidence provided in the literature, issues revolving around self-compassion, social support, and social isolation are further amplified by lack of PA and sports participation. To make matters worse, adolescent girls who are considered to be at risk may experience magnified barriers to PA and lack interest in the potential benefits of a PA program. This is problematic because this specific population may experience significant benefits from a successful PA program compared with the general population of adolescent girls. PA has been shown to positively enhance self-perceptions of self-compassion and social support and to reduce social isolation, all of which are prevalent traits among at-risk adolescent girls [7,20,44]. Therefore, there is a need for an integrated PA and psychosocial program to help address these specific issues faced by this vulnerable population, as well as to foster at-risk girls’ interests in the discussion of various psychosocial topics deemed important by them.

The GUM intervention was designed with these specific elements in mind. By providing a safe space for at-risk adolescent girls to talk about their issues and engage in fun, organized PAs that help emphasize the importance of a physically active lifestyle, the intervention’s outcomes may be highly valuable to future research. However, this study does pose some potential limitations as a result of its noncontrolled environment, the population being studied, and the constraints based on budget and resources. First, the use of subjective self-report measures may be problematic, as the ability to recall PA engagement and other psychological factors can be imperfect among adolescents [45,46]. Second, this study was exploratory in nature and did not include a control group. Therefore, a true cause and effect cannot be determined and the outcomes may have been a result of some unforeseen variables. Additionally, the outcomes from this study will provide an estimate/exploration of effectiveness immediately after the completion of the intervention and do not include long-term follow-up, making it difficult to be certain of sustained behavior change. Lastly, the fact that the GUM study was specifically targeting at-risk adolescent girls means that the findings are only generalizable and applicable to this specific population.

In conclusion, given the lack of research around at-risk adolescents, the overarching goals and objectives of the GUM program may provide the current literature with promising results and aid in the future design and refinement of effective PA programs for at-risk adolescent girls.

## Appendix

Multimedia Appendix 1 Peer Review Report Summary SSHRC CRSH.

## Abbreviations

CASSS: Child and Adolescent Social Support Scale
GoM: Girls on the Move
GUM: Girls United and on the Move
LCQ: Learning Climate Questionnaire
PA: physical activity
PHAB: Physical Health and Activity Behaviour
SCS: Self-Compassion Scale
SPIRIT: Standard Protocol Items: Recommendations for Interventional Trials
