# Supplementary material for: Exploring the Effectiveness of an Integrated Physical Activity and Psychosocial Program Targeting At-Risk Adolescent Girls: Protocol for the Girls United and on the Move (GUM) Intervention Study
Source: JMIR Res Protoc. 2020 Jun 9;9(6):e15302. doi: 10.2196/15302 (PMC7312238; doi:10.2196/15302)
Supplement: Multimedia Appendix 1 [file resprot_v9i6e15302_app1.pdf]

SSHRC CRSH

350 Albert Street, P.O Box 1610
Ottawa ON K1P 6G4
www.sshrc.ca

350, rue Albert, C.P. 1610
Ottawa ON K1P 6G4
www.crsh.ca

NOTICE OF DECISION

Applicant and Institution Information

Dr. Cristina Caperchione

Faculty of Health & Social Development
The University of British Columbia
2329 West Mall
Vancouver British Columbia V6T 1Z4

Application Information

Funding Opportunity: **Insight Development Grants**

File Number: 430-2017-00144

Scholar Type: Established

Title: A league of their own: Examining the effectiveness of a psycho-social and sport program targeting at-risk adolescent girls

Decision: **Funding Offered**

Application Results

Your scores* within committee:

Challenge (50%): 2.17
Feasibility (20%): 2.00
Capability (30%): 1.31

Total Score: 1.88
(weighted)
Overall Rating: Very Good
(weighted)

Rank: 2/11

*see scoring table in SSHRC Notes

Sextile category within overall competition:
First

Committee Results

Committee Name:
4D - Sociology, demography 2

<u>Emerging Scholars</u>
Applications: 28
Grants Offered: 9 (32.1%)
Grants Not Offered: 19 (67.9%)

Score of last funded application: 2.32

<u>Established Scholars</u>
Applications: 11
Grants Offered: 4 (36.4%)
Grants Not Offered: 7 (63.6%)

Score of last funded application: 1.99

Overall Competition Results

Applications: 1236
Grants Offered: 397 (32.12%)
Grants Not Offered: 839 (67.88%)

Sextile Categories:

First: 206 offered
Second: 191 offered / 15 not offered
Third: 206 not offered
Fourth: 206 not offered
Fifth and sixth: 412 not offered

Committee Recommendation

The committee recommended that this meritorious proposal be funded at a reduced level.

SSHRC Notes

Rating	Scoring Range
Excellent	1.00-1.83
Very Good	1.84-2.67
Good	2.68-3.50
Satisfactory	3.51-4.33
Moderate	4.34-5.16
Unsatisfactory	5.17-6.00

Note: Two rank orders were produced for each committee: one for emerging scholars and one for established scholars.

Questions? Email: insightdevelopmentgrants@sshrc-crsh.gc.ca
